# Prognostic significance of *BRCA* mutations in ovarian cancer: an updated systematic review with meta-analysis

**DOI:** 10.18632/oncotarget.12306

**Published:** 2016-09-28

**Authors:** Kai Xu, Shouhua Yang, Yingchao Zhao

**Affiliations:** ^1^ Department of Otolaryngology-Head and Neck Surgery, Tongji Hospital, Tongji Medical College, Huazhong University of Science and Technology, Wuhan, Hubei Province, China; ^2^ Department of Obstetrics and Gynecology, Union Hospital, Tongji Medical College, Huazhong University of Science and Technology, Wuhan, Hubei Province, China; ^3^ Cancer Center, Union Hospital, Tongji Medical College, Huazhong University of Science and Technology, Wuhan, Hubei Province, China

**Keywords:** ovarian cancer, BRCA mutation, prognosis, systematic review, meta-analysis

## Abstract

There is no consensus on the syntheses concerning the impact of *BRCA* mutation on ovarian cancer survival. A systematic review and meta-analysis of observational studies was conducted that evaluated the impact of *BRCA* mutations on the survival outcomes of patients with ovarian cancer. The primary outcome measure was overall survival (OS) and secondary outcome was progression-free survival (PFS). We presented data with hazard ratios (HRs) and 95% confidence interval (CI) and pooled them using the random-effects models. From 2,624 unique records, 34 eligible studies including 18,396 patients were identified. *BRCA1/2* mutations demonstrated both OS and PFS benefits in patients with ovarian cancer (OS: HR = 0.67, 95% CI, 0.57 to 0.78, I^2^ = 76.5%, *P* <0.001; PFS: HR = 0.62, 95% CI, 0.53 to 0.73, I^2^ = 18.1%, *P* = 0.261). For BRCA1 mutation carriers, the HRs for OS and PFS benefits were 0.73 (95% CI, 0.63 to 0.86) and 0.68 (95% CI, 0.52 to 0.89), respectively. For *BRCA2* mutation carriers, the HRs for OS and PFS benefits were 0.57 (95% CI, 0.45 to 0.73) and 0.48 (95% CI, 0.30 to 0.75), respectively. The results of subgroup analyses for OS stratified by study quality, tumor stage, study design, sample size, number of research center, duration of follow-up, baseline characteristics adjusted and tumor histology were mostly constant across *BRCA1/2*, *BRCA1* and *BRCA2* mutation subtypes. In summary, for patients with ovarian cancer, *BRCA* mutations were associated with improved OS and PFS. Further large-scale prospective cohort studies should be conducted to test its benefits in specific patients.

## INTRODUCTION

As two tumor suppressor genes, *BRCA1* and *BRCA2* mutation are reported to have been associated with increased risk of developing ovarian cancer and breast cancer [1–3]. Both of them are involved in DNA damage repair through homologous recombination, contributing to genomic instability and malignant transformation [4–6]. Meanwhile, they also involved in cell growth inhibition, gene transcription regulation, apoptosis and other related cellular regulation processes. Previous study reported that patients with *BRCA*-deficient ovarian cancer had improved survival rates as these patients were reported sensitive to platinum-based chemotherapy [7, 8].

Currently, numerous studies have reported the association between *BRCA* mutations and ovarian cancer mortality, and the results are conflicting. Some investigators have found that ovarian cancer patients with *BRCA* mutations have more favorable outcomes [9–18], whereas others have indicated null results [7, 19–23].

Two previous published meta-analyses have reported the prognostic impact of *BRCA* mutations on ovarian cancer mortality [24, 25]. *Sun* et al. found that patients with ovarian cancer with *BRCA* dysfunction status tended to have a better outcome [24]. However, this study investigated the effects of *BRCA* dysfunction status including mutations, protein expression and its promoter methylation, which did not perform the detailed analyses of *BRCA* mutations. In the meta-analysis by *Zhong* et al. they only examined the *BRCA1* and *BRCA2* mutation separately with limited statistical power without examining *BRCA1/2* mutation [25]. Therefore, the purpose of this study was to update the meta-analysis on the impact of *BRCA* mutation carriers versus noncarriers on mortality in patients with ovarian cancer.

## RESULTS

### Literature search and study characteristics

**Figure 1:**
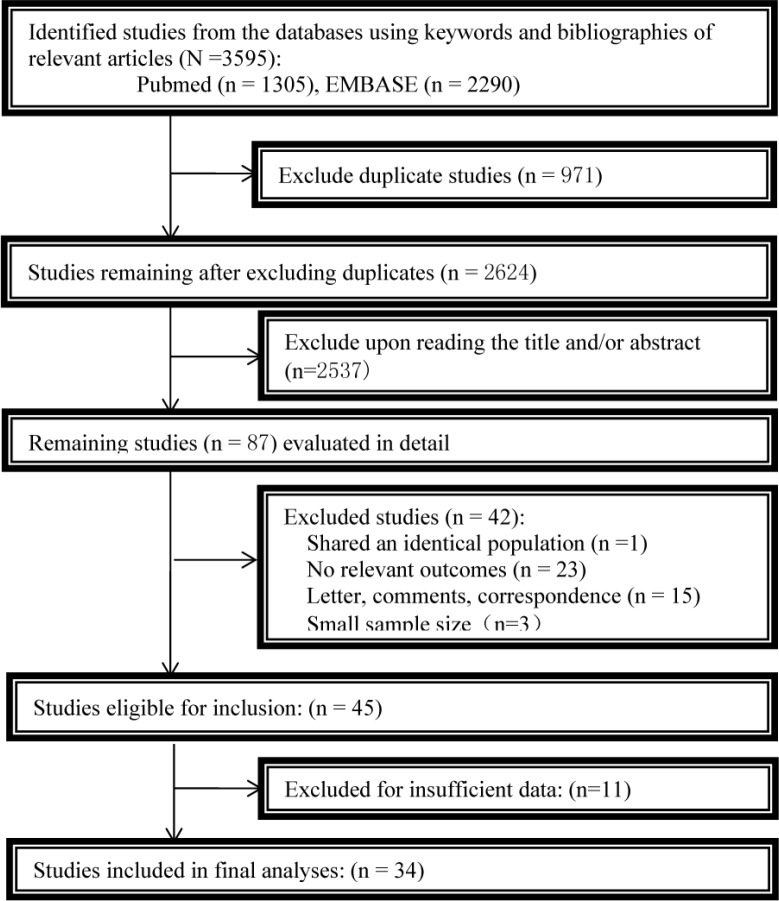
Flowchart of the study selection

From the initial literature search, we yielded 3595 citations. After exclusion of duplicate publications, 2624 citations remained for further review. 45 potentially eligible reports were selected when irrelevant studies were removed. After reading each full manuscript, we finally identified the 34 studies for meta-analysis. As is shown in Figure 1, we follow the Preferred Reporting Items for Systematic Reviews and Meta-Analyses (PRISMA) flow diagram to conduct this meta-analysis.

### Characteristics of included studies

Table 1 summarizes the baseline characteristics of the included studies. A total of 18,396 patients were included with 32 studies reporting the primary outcome of OS and 13 studies reporting the secondary outcome of PFS. *BRCA1*, *BRCA2* mutation and *BRCA1/2* mutation were reported in 15, 14 and 34 studies, respectively. All studies were published between 1996 and 2016. The mean study sample size was 541 (range 40 to 6556) with a percentage of serous cancer ranging from 24.2% to 100%. 32% (11/34) of the included study were conducted in Europe, 50% (17/34) in USA or Canada and 9% (3/34) in Asia, from which 13 were multicenter studies.

**Table 1:** Baseline Characteristics of Included Studies

Authors and published years	Study design	No. in study (cases/controls)	Inclusion period	Country of origin	Stage	Histology	serous cancer (%)	Mutation detection method	BRCA status	Germ /Soma	Single or multicenter	Follow-up Duration	Adjusted variables	Mutation ratio	Optimal debulking(%)
Synowiec (2016)	RC	17/108	2002-2008	Poland	I-IV	all	54.4	PCR, seq	BRCA1	Germ	single	NR	Age, stage, grade, histology, chemotherapy regimen, surgery, grade	13.60	61.6
Sabatier (2016)	RC	33/71	1994-2011	France	I-IV	all	52.9	MLPA, DHPLC, Seq	BRCA1/2	Germ	single	Mean 69.8 months with an s.d. of 58.4 months.	Age, stage, grade,histology, chemotherapy, surgery	31.70	67.3
Kotsopoulos (2016)	RC	177/1244	1995-1999, 2002-2004	Canada	I-IV	all	55.1	NR	BRCA1/2	NR	single	BRCA1 Mutation: 8.1 years (range0.94–20.2) BRCA2 Mutation: 7.6 years (range 2.08–19.9) WT: 9.7 years (range 0.59–20.3)	Age, stage, grade, histology, surgery	12.50	NR
Harter (2015)	RC	97/567	NR	Germany	II-IV	all	73.6	PCR, seq	BRCA1/2	Germ	single	NR	Age, stage, grade, histology	15	NR
Chen (2015)	RC	61/195	NR	Finland	NR	NR	NR	PCR, Seq	BRCA1/2	NR	multicenter	NR	Age, grade, stage, residual disease, neoadjuvant therapy, primary therapy outcome	23.82	NR
Candido-dos-Reis (2015)	RC	1496/5060	NR	U.S.A	I-IV	all	NR	PCR, Seq	BRCA1/2	Germ	multicenter	NR	Stage, regional, and distant, histology, grade,	22.80	NR
Cunningham (2014)	RC	70/993	1992-2011	U.S.A	I-IV	all	73	PCR, seq	BRCA1/2	Germ+Soma	single	Median 4.5 years (range 0.01-10)	Age, stage, grade, debulking status, ascites present at surgery, menopausal status	6.6	85
Zhang (2014)	RC	75/250	2012	NR	II-IV	NR	NR	PCR, seq	BRCA1/2	NR	single	NR	Age, grade, stage, residual tumor size, response to chemotherapytherapy	23.10	67.1
Rudaitis (2014)	RC	55/52	2008-2011	Lithuania	III-IV	nonmucinous	92.5	PCR, seq	BRCA1/2	Germ	single	BRCA1/2 Mutation: Median 35 months (range 1-169); BRCA1/2 WT; Median 25 months (range 8-210)	Age,follow-up, ECOG, Histology, subtype, residual tumor size, neoadjuvant therapy, Family history	51.40	NR
Pennington (2014)	RC	91/276	NR	U.S.A	I-IV	all	70.3	PCR, seq	BRCA1/2	Germ+Soma	multicenter	NR	Age, site, grade, stage, residual tumor size	24.8	66
Safra (2013)	RC	90/100	1995-2009	U.S.A, Israeli, Italia	I-IV	all	69.5	PCR, Seq	BRCA1/2	Germ	multicenter	Median 56 months (range 9.3–214)	Age, stage, residual tumor size, Ethnicity, Institution	47.40	NR
McLaughlin (2013)	RC	218/1408	1995-1999, 2002-2004	Canada	I-IV	all	55.8	PTT, DGGE, DHPLC, seq	BRCA1/2	Germ	multicenter	Mean 6.9 years (range 0.3-15.7)	Age, histology, grade, stage	13.40	NR
Hyman (2012)	RC	47/143	1996-2011	U.S.A	III-IV	serous	100	PCR, seq	BRCA1/2	Germ	single	Median 2.5 years	Age, stage, Optimal debulking, IP/IV	24.70	76.3
Dann (2012)	RC	15/38	1999-2007	U.S.A	II-IV	all	73.6	PCR, seq	BRCA1/2	Germ+Soma	single	NR	Age, grade, stage, histology, residual disease, chemotherapy, Platinum response	28.30	83
Chan (2012)	RC	69/246	NR	U.S.A	II-IV	serous	100	PCR, seq	BRCA1/2	Germ+Soma	single	Median 35.4 months (range 1–125)	Age, grade, stage, histology, residual disease, Ethnicity	21.80	65.8
Alsop (2012)	RC	141/860	2002-2006	Australian	I-IV	all	70.8	PCR, seq	BRCA1/2	Germ	single	Median 63.4 months	Age, stage, grade, debulking, primary site, chemotherapy, Ethnicity	14.10	62.4
Yang (2011)	RC	62/252	2009-2010	U.S.A	II-IV	serous	100	PCR, seq	BRCA1/2	Germ+Soma	single	NR	Age, stage, grade, debulking, Ethnicity	19.70	65.8
Lacour (2011)	RC	95/183	1996-2007	U.S.A	III-IV	all	68	PCR, seq	BRCA1/2	NR	multicenter	BRCA Mutation; median 42.6 ; BRCA WT; median 37.5	Age, stage, grade, histology, debulking, response to chemotherapy, Institution, Ethnicity	34.20	70.1
Gallagher (2011)	RC	36/74	1996-2006	U.S.A	III-IV	all	80.9	PCR, Seq	BRCA1/2	Germ	single	Median 41 months	Age, stage, histology, debulking, platinum response, CA125, secondary cytoreduction	32.70	60.9
Hennessy (2010)	RC	44/191	1996-2006	U.S.A	I-IV	all	79.1	PCR,Seq	BRCA1/2	Germ+Soma	multicenter	Median 1071days (range 19-6241)	Age, grade, stage, residual disease, surgery, chemotherapy	18.70	58.7
Tan (2008)	NCC	22/44	1993-1995	UK	II-IV	all	81.8	SCCP, seq	BRCA1/2	Germ	multicenter	NR	Age, stage, histology	33.30	NR
Chetrit (2008)	RC	225/554	1994-1999	Israel	I-IV	all	57.1	PCR, seq	BRCA1/2	Germ	single	Median 6.2 years (range 4.2-9.4)	Age, stage, grade, menopausal status	28.90	NR
Pal (2007)	RC	32/200	2000-2003	U.S.A	I-IV	all	57.9	PCR, seq	BRCA1/2	Germ	single	NR	Age, grade, stage, histology	13.80	NR
Majdak (2005)	NCC	18/187	1994-2002	Poland	I-IV	all	64.9	F-CSGE, PCR, Seq	BRCA1/2	Germ	single	NR	Age, stage, grade, histology, residual, outcome, Infertility	8.8	52.2
Cass (2003)	RC	34/37	1990-1998	U.S.A	I-IV	all	84.5	PCR, SSCR, seq	BRCA1/2	Germ	single	Median 72months	Age, stage, grade, histology, CA125, optimal cytoreduction, Primary chemotherapy,	47.90	NR
David (2002)	RC	234/662	1994-1999	Israel	NR	all	NR	SCCP,seq	BRCA1/2	Germ	single	Median 30.5 months(range 20-64)	age, stage, family history	26.10	NR
Buller (2002)	NCC	24/48	NR	U.S.A	I-IV	all	74.6	PCR,PTT, seq, SSCP	BRCA1	Germ+Soma	single	NR	/	23.60	NR
Zweemer (2001)	NCC	23/17	NR	Netherland	I-IV	all	55	PCR,PTT	BRCA1/2	Germ	single	Mean 47 months (range 6-168)	age, stage, grade	57.50	NR
Ramus (2001)	RC	27/71	1992-1997	Israel	I-IV	all	77.6	RCR, SSCP, seq	BRCA1/2	Germ	single	NR	/	27.60	NR
Boyd (2000)	RC	88/101	1986-1998	U.S.A	I-IV	all	64	PCR, Seq	BRCA1/2	Germ	multicenter	BRCA Mutation; median 57 months; BRCA WT; median 59 months	Histology, grade, stage, cytoreductive surgery, chemotherapy	46.60	50.8
Pharoah (1999)	NCC	38/127	NR	UK	I-IV	all	24.2	PTT, SSCP, Seq	BRCA1/2	Germ	multicenter	NR	/	56	NR
Johannsson (1998)	NCC	38/97	1985-1995	Sweden	I-IV	all	NR	PTT, SSCP, Seq	BRCA1	Germ	multicenter	NR	/	28.15	NR
Aida (1998)	NCC	13/49	1983-1997	Japan	III	all	83.9	SSCP, PCR, Seq	BRCA1	Germ	multicenter	Mean 54.8 months	Age, histology, stage, chemotherapy, response,	57.80	NR
Rubin (1996)	NCC	43/43	1998-1996	U.S.A	III-IV	all	81.1	SSCP, PCR, Seq	BRCA1	Germ	multicenter	Mean 71 months	/	50	NR

Abbreviations: DGGE = fluorescent multiplex denaturing gradient gel electrophoresis; DHPLC = Denaturing high performance liquid chromatography; F-CSGE = Fluorescence-based Conformation Sensitive Gel Electrophoresis; Germ = germline mutation; IP= combined intravenous and intraperitoneal therapy; IV= intravenous therapy alone; MLPA = multiplex ligation-dependent probe amplification; NCC=Nested case-control study; NR=not reported; PTT = Protein truncation test; RC=retrospective cohort; RFLP = Restriction fragment length polymorphisms; seq = sequencing; Soma = somatic mutation; SSCP = Single-Strand Conformation Polymorphism;

As shown in Supplementary Table S1, the quality of the 34 included studies was generally high with 17 studies being more than 7 points.

### Survival analysis for *BRCA1/2*-mutation carriers with ovarian cancer

#### OS analysis

**Figure 2:**
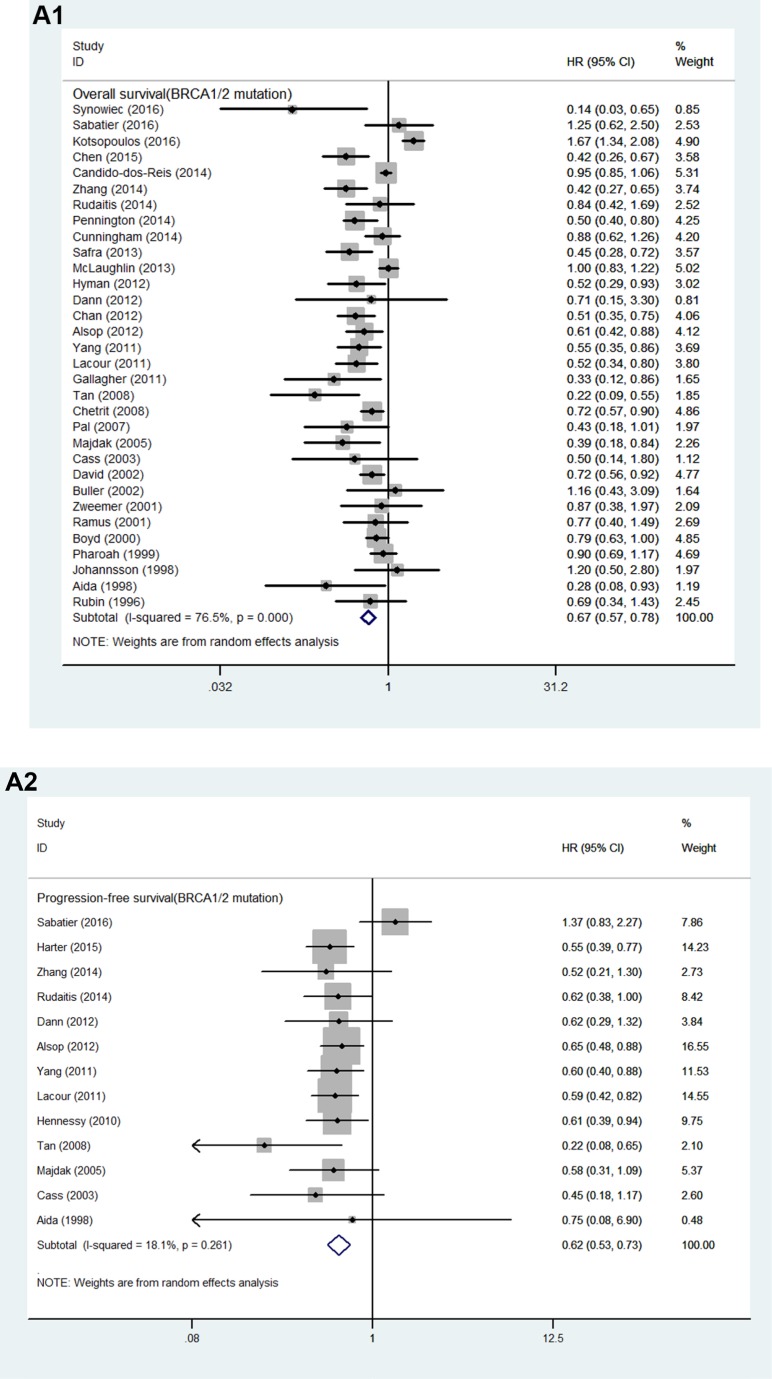

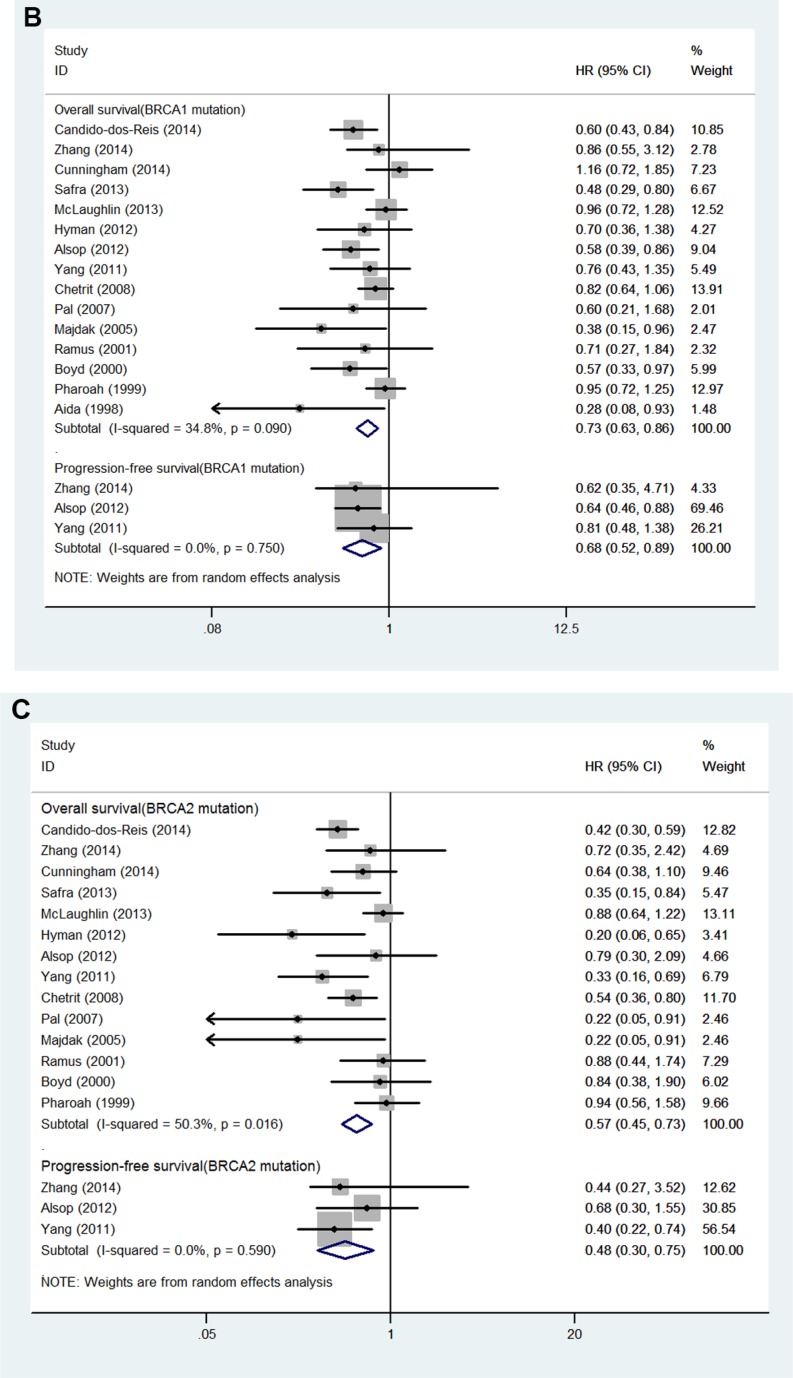
(**A**) Forest plot for the association between *BRCA1/2* mutation and ovarian cancer (1) overall survival and (2) progression-free survival. (**B**) Forest plot for the association between *BRCA1* mutation and ovarian cancer overall survival and progression-free survival; (**C**) Forest plot for the association between *BRCA2* mutation and ovarian cancer overall survival and progression-free survival.

32 studies of 17,497 patients with either *BRCA1* or *BRCA2*-mutation (*BRCA1/2*-mutation) were identified in this analysis. Patients with *BRCA1/2*-mutation had significant OS benefit (HR = 0.67, 95% CI, 0.57 to 0.78, I^2^ = 76.5%, *P* < 0.001; Figure 2A).

Subgroup analyses revealed that studies with adequate adjusted variables, but not with inadequate adjusted variables had statistically significant OS benefit in ovarian cancer patients with *BRCA1/2*-mutation (adequate adjusted variables, HR = 0.63, 95% CI, 0.53 to 0.75, I^2^ = 80.7%, *P* < 0.001; inadequate adjusted variables, HR = 0.89, 95% CI, 0.72 to 1.10, I^2^ = 0, *P* = 0.992). OS benefits were also indicated in other subgroups and the HRs for all of the different subgroups are summarized in Table 2A.

**Table 2A:** Subgroup analyses stratified by some of the baseline characteristics for associations between *BRCA1/2* mutation and overall survival

	HR	95%CI	Degree of heterogeneity (I^2^ statistics; %)	*P*	No. of included Studies
Total					
	0.67	0.57 to 0.78	76.5	< 0.001	32
Study quality					
Score > 7	0.67	0.56 to 0.80	74.4	< 0.001	15
≤ 7	0.66	0.50 to 0.87	79.2	< 0.001	17
Stage of disease					
I-IV	0.79	0.66 to 0.94	76.5	< 0.001	19
II-IV	0.47	0.37 to 0.59	0	0.423	5
III-IV	0.64	0.49 to 0.83	43.2	< 0.091	8
Study design					
Cohort	0.67	0.56 to 0.79	80.1	< 0.001	24
Case-control	0.65	0.44 to 0.96	56.7	0.024	8
Sample size					
≥ 200	0.68	0.56 to 0.83	85.5	< 0.001	5
< 200	0.67	0.54 to 0.83	45.7	0.021	17
Research center					
Single	0.67	0.53 to 0.84	76.7	< 0.001	20
Multicenter	0.70	0.57 to 0.86	75.5	< 0.001	11
Duration of follow-up Months					
> 60	0.77	0.65 to 0.91	77.7	< 0.001	20
≤ 60	0.58	0.49 to 0.68	40.7	0.069	12
Adequate baseline characteristics adjusted					
Yes	0.63	0.53 to 0.75	80.7	< 0.001	26
No	0.89	0.72 to 1.10	0	0.922	6
Histology					
All	0.68	0.58 to 0.79	76.2	< 0.001	31
High-grade serous	0.62	0.43 to 0.90	84	< 0.001	4
Mutation ratio					
> 25%	0.70	0.60 to 0.81	36.3	0.068	17
≤ 25%	0.65	0.51 to 0.82	85.5	< 0.001	15
Region					
Europe	0.65	0.48 to 0.88	64.8	0.002	10
America/Canada	0.72	0.59 to 0.89	80.3	< 0.001	16
Asia	0.69	0.51 to 0.93	12.0	0.321	3
Optimal debulking ratio					
> 65%	0.58	0.48 to 0.72	41.6	0.090	9
≤ 65%	0.53	0.35 to 0.79	60.9	0.037	5

Abbreviations: HR = hazard ratio; CI = confidence interval.

**Table 2B:** Subgroup analyses stratified by some of the baseline characteristics for associations between *BRCA1* mutation and overall survival

	HR	95%CI	Degree of heterogeneity (I^2^ statistics; %)	*P*	No. of included Studies
Total	0.73	0.63 to 0.86	34.8	0.090	15
Study quality					
Score > 7	0.76	0.63 to 0.91	35.2	0.137	9
≤ 7	0.66	0.47 to 0.91	44.6	0.108	6
Stage of disease					
I–IV	0.72	0.58 to 0.88	47.1	0.057	9
II–IV	0.79	0.49 to 1.27	0	0.816	2
III–IV	0.69	0.46 to 1.04	50.2	0.110	4
Study design					
Cohort	0.73	0.63 to 0.86	22.6	0.221	12
Case-control	0.54	0.24 to 1.24	69.9	0.036	3
Sample size					
≥ 200	0.76	0.64 to 0.92	33.3	0.152	9
< 200	0.65	0.47 to 0.90	46.2	0.098	6
Research center					
Single	0.77	0.65 to 0.90	0	0.461	9
Multicenter	0.69	0.52 to 0.91	63.6	0.017	6
Duration of follow-up Months					
> 60	0.74	0.61 to 0.88	51.5	0.029	10
≤ 60	0.68	0.47 to 0.99	0	0.645	5
Adequate baseline characteristics adjusted					
Yes	0.70	0.59 to 0.83	35.5	0.099	13
No	0.93	0.71 to 1.21	0	0.568	2
Histology					
All	0.70	0.59 to 0.83	35.5	0.099	13
High-grade serous	0.78	0.53 to 1.14	74.0	0.009	4
Region					
Europe	0.82	0.64 to 1.06	44.3	0.166	3
America/Canada	0.77	0.62 to 0.96	31.2	0.190	7
Asia	0.48	0.20 to 1.19	27.1	0.242	2

Abbreviations: HR=hazard ratio; CI= confidence interval.

**Table 2C:** Subgroup analyses stratified by some of the baseline characteristics for associations between *BRCA2* mutation and overall survival

	HR	95%CI	Degree of heterogeneity (I^2^ statistics; %)	*P*	No. of included Studies
Total	0.57	0.45 to 0.73	50.3	0.016	14
Study quality					
Score > 7	0.53	0.40 to 0.70	50.2	0.034	10
≤ 7	0.71	0.41 to 1.21	46.7	0.131	4
Stage of disease					
I–IV	0.56	0.43 to 0.74	39.6	0.103	9
II–IV	0.46	0.21 to 0.97	37.2	0.207	2
III–IV	0.64	0.32 to 1.28	64.0	0.062	3
Study design					
Cohort	0.56	0.43 to 0.72	48.3	0.030	12
Case-control	0.54	0.13 to 2.14	70.7	0.065	2
Sample size					
≥ 200	0.54	0.41 to 0.73	51.4	0.036	9
< 200	0.63	0.38 to 1.04	52.9	0.075	5
Research center					
Single	0.52	0.39 to 0.70	24.6	0.224	9
Multicenter	0.65	0.43 to 0.89	71.5	0.007	5
Duration of follow-up Months					
> 60	0.59	0.44 to 0.78	55.8	0.016	10
≤ 60	0.52	0.28 to 0.94	45.4	0.139	4
Adequate baseline characteristics adjusted					
Yes	0.52	0.40 to 0.68	48.7	0.029	12
No	0.92	0.61 to 1.39	0	0.881	2
Histology					
All	0.56	0.44 to 0.72	36.7	0.097	12
High-grade serous	0.54	0.32 to 0.93	79.8	0.002	4
Region					
Europe	0.61	0.34 to 1.07	59.0	0.087	3
America/Canada	0.51	0.34 to 0.76	66.5	0.006	7
Asia	0.88	0.44 to 1.75	/	/	1

Abbreviations: HR = hazard ratio; CI = confidence interval.

#### PFS analysis

We identified 13 studies involving 3,485 patients with *BRCA1/2*-mutation for analysis of PFS [7, 10, 13, 20, 26–34]. Patients with *BRCA1/2*-mutation had significant PFS benefit (HR = 0.62, 95% CI, 0.53 to 0.73, I^2^ = 18.1%, *P* = 0.261; Figure 2A). The results of subgroup analyses for the association between *BRCA1/2*-mutation and PFS are demonstrated in Table 3A. In summary, *BRCA1/2*-mutation was significantly associated with improved PFS for studies stratified according to study quality, study design, number of research center, tumor histology and study region. The trend toward an improved PFS was also observed when studies were stratified by tumor stage, sample size, duration of follow-up and optimal debulking ratio.

**Table 3A:** Subgroup analyses stratified by some of the baseline characteristics for associations between *BRCA1/2* mutation and progression-free survival

	HR	95%CI	Degree of heterogeneity (I^2^ statistics; %)	*P*	No. of included Studies
Total					
	0.62	0.53 to 0.73	18.1	0.261	13
Study quality					
Score > 7	0.65	0.52 to 0.81	40.9	0.118	7
≤ 7	0.59	0.46 to 0.75	0	0.523	6
Stage of disease					
I–IV	0.74	0.47 to 1.15	63.0	0.044	4
II–IV	0.55	0.43 to 0.69	0	0.520	5
III–IV	0.60	0.48 to 0.76	0	0.996	4
Study design					
Cohort	0.64	0.55 to 0.74	15.9	0.296	10
Case-control	0.44	0.22 to 0.86	4	0.271	3
Sample size					
≥ 200	0.61	0.51 to 0.72	0	0.996	6
< 200	0.62	0.37 to 1.04	60.7	0.026	6
Research center					
Single	0.65	0.54 to 0.78	24	0.230	9
Multicenter	0.56	0.42 to 0.75	10.7	0.340	4
Duration of follow-up Months					
> 60	0.60	0.30 to 1.18	74.6	0.005	4
≤ 60	0.60	0.51 to 0.70	42.2	0.995	7
Adequate baseline characteristics adjusted					
Yes	0.62	0.53 to 0.73	18.1	0.261	13
No	/	/	/	/	0
Histology					
All	0.64	0.52 to 0.78	28.4	0.175	11
High-grade serous	0.60	0.40 to 0.89	/	/	1
Mutation ratio					
> 25%	0.60	0.51 to 0.71	0	0.987	6
≤ 25%	0.63	0.43 to 0.92	55.6	0.036	7
Region					
Europe	0.63	0.40 to 0.98	70.7	0.008	5
America/Canada	0.59	0.48 to 0.73	0	0.985	5
Asia	0.75	0.08 to 6.90	/	/	1
Optimal debulking ratio					
> 65%	0.70	0.50 to 1.00	55.1	0.063	5
≤ 65%	0.63	0.50 to 0.79	0	0.938	3

Abbreviations: HR = hazard ratio; CI = confidence interval.

**Table 3B:** Subgroup analyses stratified by some of the baseline characteristics for associations between *BRCA1* mutation and progression-free survival

	HR	95%CI	Degree of heterogeneity (I^2^ statistics; %)	*P*	No. of included Studies
Total					
	0.68	0.52 to 0.89	0	0.750	3
Study quality					
Score > 7	0.78	0.48 to 1.27	0	0.709	2
≤ 7	0.64	0.46 to 0.88	/	/	1
Stage of disease					
I–IV	0.64	0.46 to 0.88	/	/	1
II–IV	0.78	0.48 to 1.27	0	0.709	2
III–IV	/	/	/	/	0
Study design					
Cohort	0.68	0.52 to 0.89	0	0.750	3
Case-control	/	/	/	/	0
Sample size					
≥ 200	0.68	0.52 to 0.89	0	0.750	3
< 200	/	/	/	/	0
Research center					
Single	0.68	0.52 to 0.89	0	0.750	3
Multicenter	/	/	/	/	0
Duration of follow-up Months					
> 60	0.64	0.46 to 0.88	/	/	1
≤ 60	0.78	0.48 to 1.27	0	0.709	2
Adequate baseline characteristics adjusted					
Yes	0.68	0.52 to 0.89	0	0.750	3
No	/	/	/	/	0
Histology					
All	0.70	0.59 to 0.83	35.5	0.099	2
High-grade serous	0.81	0.48 to 1.37	/	/	1
Region					
Europe	/	/	/	/	0
America/Canada	0.81	0.48 to 1.38	/	/	1
Asia	/	/	/	/	0

Abbreviations: HR = hazard ratio; CI = confidence interval.

**Table 3C:** Subgroup analyses stratified by some of the baseline characteristics for associations between *BRCA2* mutation and progression-free survival

	HR	95%CI	Degree of heterogeneity (I^2^ statistics; %)	*P*	No. of included Studies
Total					
	0.48	0.30 to 0.75	0	0.590	3
Study quality					
Score > 7	0.41	0.24 to 0.70	0	0.895	2
≤ 7	0.68	0.30 to 1.55	/	/	1
Stage of disease					
I–IV	0.68	0.30 to 1.55	/	/	1
II–IV	0.41	0.24 to 0.70	0	0.895	2
III–IV	/	/	/	/	0
Study design					
Cohort	0.48	0.30 to 0.75	0	0.590	3
Case-control	/	/	/	/	0
Sample size					
≥ 200	0.48	0.30 to 0.75	0	0.590	3
< 200	/	/	/	/	0
Research center					
Single	0.48	0.30 to 0.75	0	0.590	3
Multicenter	/	/	/	/	0
Duration of follow-up Months					
> 60	0.68	0.30 to 1.55	/	/	1
≤ 60	0.41	0.24 to 0.70	0	0.895	2
Adequate baseline characteristics adjusted					
Yes	0.48	0.30 to 0.75	0	0.590	3
No	/	/	/	/	0
Histology					
All	0.40	0.22 to 0.73	/	/	1
High-grade serous	0.60	0.30 to 1.20	0	0.576	2
Region					
Europe	/	/	/	/	0
America/Canada	0.40	0.22 to 0.74	/	/	1
Asia	/	/	/	/	0

Abbreviations: HR = hazard ratio; CI = confidence interval.

**Figure 3:**
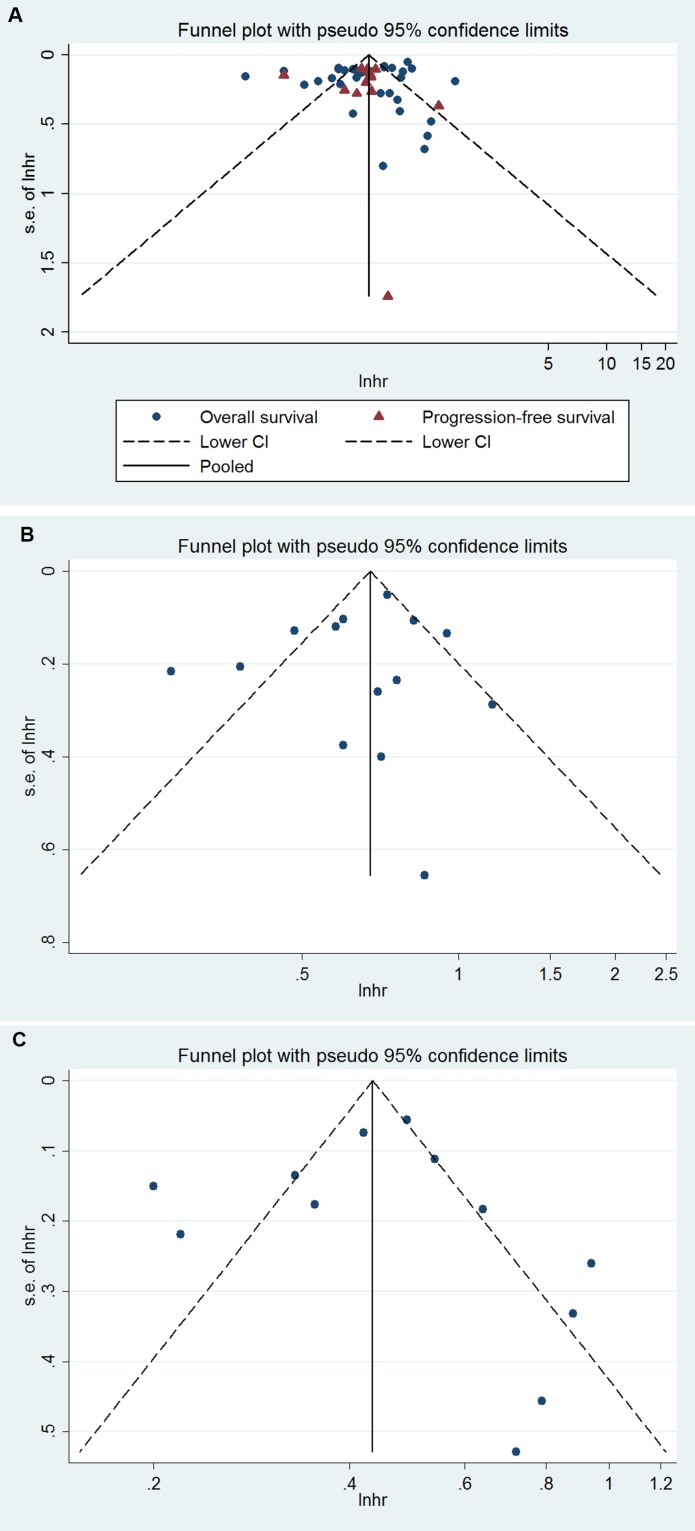
Funnel plot for (**A**) *BRCA1/2*, (**B**) *BRCA1,* (**C**) *BRCA2* mutation and ovarian cancer overall survival and/or progression-free survival

No evident publication bias was observed by funnel plot asymmetry (Figure 3A) or through Begg's test (OS, *P* = 0.72; PFS, *P* = 0.58) or Egger's test (OS, *P* = 0.23; PFS, *P* = 0.93). The trim and fill method applied to further conduct the sensitivity analysis indicated 8 and 5 missing studies in the funnel plot for OS and PFS, respectively (Figure 3). However, imputing these hypothesized studies did not substantially alter the primary pooled estimates (OS, adjusted HR = 0.49, 95% CI 0.41 to 0.59; PFS, adjusted HR = 0.48, 95% CI 0.40 to 0.58).

### Survival analysis for *BRCA1*-mutation carriers with ovarian cancer

#### OS analysis

15 studies involving 12,995 patients with *BRCA1*-mutation were identified for meta-analysis [12, 13, 27, 28, 32, 34–43]. Patients with *BRCA1*-mutation had significant OS benefit (HR = 0.73, 95% CI, 0.63 to 0.86, I^2^ = 34.8%, *P* < 0.001; Figure 2B).

The results of subgroup analyses for the association between *BRCA1*-mutation and PFS are presented in Table 2B. We found that ovarian cancer patients with *BRCA1*-mutation had significantly longer OS than non-carriers, regardless of study quality, sample size, research center or duration of follow-up. Such trend was also noted in studies with cohort study design, adequate baseline characteristics adjusted, all histologic types or conducted in USA or Canada.

#### PFS analysis

We identified 3 studies involving 1,640 patients with *BRCA1*-mutation for analysis of PFS [13, 27, 28]. Patients with *BRCA1*-mutation had significant PFS benefit (HR = 0.68, 95% CI, 0.52 to 0.89, I^2^ = 0, *P* < 0.001; Figure 2B). The results of subgroup analyses for the association between *BRCA1*-mutation and PFS are demonstrated in Table 3B.

No evident publication bias was observed by funnel plot asymmetry (Figure 3B) or through Egger's test (*P* = 0.84) or Begg's test (*P* = 0.83) for OS. The trim and fill method applied to further conduct the sensitivity analysis indicated one missing study in the funnel plot for OS (Figure 3). However, imputing this hypothesized study did not alter the primary pooled estimates (adjusted HR = 0.66, 95 % CI, 0.56 to 0.78). We did not investigate the publication bias for PFS due to the limited number of studies.

### Survival analysis for *BRCA2*-mutation carriers with ovarian cancer

#### OS analysis

14 studies including 12,933 patients with *BRCA2*-mutation were involved for meta-analysis [12, 13, 27, 28, 32, 35–43]. Patients with *BRCA2*-mutation had significant OS benefit (HR = 0.57, 95% CI, 0.45 to 0.73, I^2^ = 50.3%, *P* < 0.001; Figure 2C).

The results of subgroup analyses for the association between *BRCA2*-mutation and OS are presented in Table 2C. We found that ovarian cancer patients with *BRCA2*-mutation had significantly longer OS than non-carriers, regardless of research center, duration of follow-up or histologic type. Such trend was also noted in studies with high quality, II-IV disease stage, cohort study design, sample size larger than 200, adequate baseline characteristics adjusted or conducted in USA or Canada.

#### PFS analysis

We identified 3 studies involving 1,640 patients with *BRCA2*-mutation for analysis of PFS [13, 27, 28]. Patients with *BRCA2*-mutation had significant PFS benefit (HR = 0.48, 95% CI, 0.30 to 0.75, I^2^ = 0, *P* < 0.001; Figure 2C). The results of subgroup analyses for the association between *BRCA2*-mutation and PFS are demonstrated in Table 3C.

No evident publication bias was observed by funnel plot asymmetry (Figure 3C) or through Egger's test (*P* = 0.54) or Begg's test (*P* = 0.96) for OS. The trim and fill method applied to further conduct the sensitivity analysis indicated 4 missing studies in the funnel plot for OS (Figure 3). However, imputing these hypothesized studies did not alter the primary pooled estimates (adjusted HR = 0.38, 95 % CI 0.29 to 0.50). We did not investigate the publication bias for PFS due to the limited number of studies.

## DISCUSSION

The aim of this meta-analysis was to examine the association between *BRCA* mutation status and ovarian cancer survival (OS and PFS). By pooling the outcomes of 18,396 ovarian cancer patients from 34 individual studies, we found that *BRCA* mutation (*BRCA1/2*, *BRCA1* and *BRCA2*) carriers had significantly improved OS and PFS benefits in ovarian cancer patients. Subgroup analysis revealed that this survival benefits remained constant irrespective of study quality, tumor stage, study design, sample size, number of research center, duration of follow-up, baseline characteristics adjusted and tumor histology.

This meta-analysis showed that patients who were *BRCA* mutation carriers had a 33%, 27% and 43% reduction in all-cause mortality for *BRCA1/2*, *BRCA1* and *BRCA2* mutants respectively, while patients had a 38%, 32% and 52% reduction in progression-free mortality for *BRCA1/2*, *BRCA1* and *BRCA2* mutants, respectively. Individually, however, some of the studies had reported contradictory findings [7, 19–22]: some studies have indicated significantly reduced all-cause mortality or progression-free mortality among *BRCA* mutation carriers [9–14], whereas *Kotsopoulos* et al. reported that the mortality risk in ovarian cancer patients was significantly poorer for *BRCA* mutation carriers than for non-carriers (HR = 1.67; 95% CI 1.34 to 2.08) [19]. The present meta-analysis with the largest number of patients investigated both survival (OS) and progression outcomes (PFS) for ovarian cancer, incorporating not only the general *BRCA* mutation status but also two subtypes, including *BRCA1* and *BRCA2* mutation status.

It has been reported that germline *BRCA1/2* mutations occur in approximately 10 to 20% of patients with invasive epithelial ovarian cancers [7, 9–14, 19–22], and more than 20% of patients with high-grade serous ovarian cancer [12]. *BRCA1/2* tumor suppressor genes are reported to be involved in DNA repair through homologous recombination, through which pathway genes are unable to repair DNA double-strand, resulting in genomic instability and having a tendency to malignant transformation [3]. On the other hand, the impairment of this pathway can also influence DNA cross-links by tumor cells, which can be induced by cisplatin, a chemotherapy agent for ovarian cancer. It has been indicated that *BRCA*-deficient patients can have better survival outcomes through the increase in the response rate to platinum-based chemotherapy [7, 8].

The findings of this updated meta-analysis are generally consistent with and further extend the other two published systematic reviews and meta-analyses in several important ways. First, our study had added greater statistical power to the associations between *BRCA* mutations and ovarian cancer survival with more detailed subgroup analyses. For example, the present meta-analysis involved approximately 2.3 times as many participants as the previous two studies [24, 25]. As a matter of fact, 11 recent published cohort studies were involved in the analyses. Second, three mutation subtypes (*BRCA1/2*, *BRCA1* and *BRCA2*) were thoroughly investigated in contrast to the earlier two meta-analyses (including only *BRCA1* and *BRCA2* subtypes). Thirdly, we did detailed subgroup analyses under a broader range of study level circumstances to examine the potential sources of heterogeneity. However, our findings concur with the previous meta-analyses. Inter-study heterogeneity was found very high for a number of analyses, which was probably due to the very variation in population characteristics and *BRCA* mutation detection methods.

Thus, caution is required when interpreting these findings. Moreover, one important advantage of this meta-analyses lies in that we have thoroughly tested the influence of publication bias through Begg's test, Egger's test and sensitivity analysis and confirmed the robustness of the findings.

Several limitations of this meta-analysis are required to be addressed. We acknowledge that the results of this meta-analysis were derived from published data rather than from studies of individual patient data. Thus, we could not obtained the detailed characteristics of each individual from the involved studies including patient age, tumor stage, sample size, and follow-up period, which to some extent were contributory factors to the heterogeneity, but an attempt was made to account for this variation by conducting subgroup analyses. Another potential limitation of the study is that we also include some conference abstracts in the analysis. It is likely that the results may differ to certain extent between the conference abstracts and future updated full publication. However, we proposed that such differences are very likely to be relatively mild. Moreover, the method for the detection of *BRCA* mutation varied among studies, which may also a source of substantial heterogeneity. Some of the included studies did not report complete data for analysis, and could have potentially affected the results of multivariate analysis. Most of the included studies adequately adjusted for some known confounders, in particular patients age, tumor stage and grade or chemotherapy. However, some studies did not assess these factors and we acknowledge this limitation. As these were all studies with small sample size, it is unlikely to have affected the results of the analysis substantially. Although no obvious evidence of publication bias was noted in each subset of meta-analysis, it was still a major concern. Due to the time taken to conduct this meta-analysis, further relevant studies concerning this topic may have been published. However, given the relative paucity of suitable studies identified through the last 20 years from 1996 to 2016, we proposed that there were probably very few studies in number and they would not substantially affect the general conclusions of this study.

Despite all of these limitations, however, our meta-analysis with a large sample size of over 18,396 participants, and used the appropriate analyses to investigate the heterogeneity and publication bias among the different studies, showed that in patients with ovarian cancer, *BRCA* mutation (irrespective of its subtypes) carriers had better OS and PFS than non-carriers. Whether the results may have therapeutic implications remains to be elucidated with further larger, well-designed studies in specific ovarian cancer patients.

## MATERIALS AND METHODS

### Literature search and study selection

PubMed and EMBASE were searched for studies published up to February 2016 for the following searching terms: (ovary/ovarian/oophor* and cancer/neoplas*/tumor*/tumour*/ cancer*/carcinoma*/malignan*/neoplasms) and (*BRCA1/2* and mutation*/mutated) and mortality/survival/prognosis. Mesh (Pubmed) and Emtree (Embase) terms combined with free text words were used for searching. Detailed search terms and strategies for the two databases are presented in Supplementary Appendix 1. In addition, we also conducted the manual searches of references in all eligible studies to identify potential missing publications that were not identified during the preliminary literature searches. We did not place any restrictions on the searches.

Studies were considered eligible if they met the following inclusion criteria: observational studies (cohort or case-control studies) that investigated patients with ovarian cancer assessed for *BRCA* mutation status (*BRCA1* or *BRCA2* mutation status). The outcome measures included OS and PFS, measured as the relative risk (RR), the odds ratio (OR), or the hazard ratio (HR) along with the 95% confidence interval (CI) (or sufficient data for calculating them). We did not include studies with unpublished data. If multiple reports contained the duplicated datasets, the report with the largest or the most recent data was included for analysis. Two investigators independently conducted the literature review (KX and SHY) and any discrepancies were resolved by discussion or by a senior investigator (YCZ).

### Data extraction and quality assessment

Two investigators independently extracted data from each included study using a predefined standardized data extraction form including the pertinent issues that concerned the characteristics and survival outcomes of the ovarian cancer patients. For each article, the following information was extracted: authors and published years, study design, sample size, inclusion period, research country, disease stage, tumor histology, *BRCA* mutation detection methods, research center involved, duration of follow-up and adjusted variables. The extracted data were crosschecked and any disagreements were resolved by discussion.

### Outcome measures

The primary outcome measure was OS defined as the time from initial ovarian cancer diagnosis to death due to any causes. Secondary outcome was PFS defined as the time from diagnosis to the first confirmed sign of cancer recurrence, or progression (disease relapse or metastasis) or death from any cause.

### Quality assessment

The nine-star Newcastle-ottawa Scale (NOS) [44] was used to assess the study quality for each study. Three domains associated with the selection of study population, data comparability and exposure (case-control studies) or outcome (cohort studies) assessment were evaluated. The NOS score ranged from 0 to 9 with a score > 7 indicating high quality. Two investigators scored each study, and any discrepancies were resolved by a third investigator.

### Statistical analysis

All statistical analyses were performed using Stata statistical software (version 12.0; Stata Corporation, College Station, TX, USA). Pooled HRs for OS and PFS with 95% CIs were calculated using random-effects model due to the potential substantial heterogeneity between studies [45]. Heterogeneity across studies was examined by I^2^ statistic with an I^2^ ≥ 50% indicating the presence of significant heterogeneity [46]. We further investigated potential heterogeneity by subgroup analyses stratified by study quality, tumor stage, study design, sample size, number of research center, duration of follow-up, baseline characteristics adjusted, mutation ratio and tumor histology for OS and PFS across *BRCA1/2*, *BRCA1* and *BRCA2* mutation subgroups. Publication bias was evaluated by observing the asymmetry of funnel plots and using the Begg-Mazumdar rank correlation test and Egger's test [47, 48]. The Duval and Tweedie trim-and-fill method was also applied to conduct sensitivity analysis [49]. A two-sided *P* ≤ 0.05 was considered statistically significant.

## SUPPLEMENTARY MATERIALS



## Abbreviations

CI: 95% confidence interval
HR: hazard ratios
NOS: Newcastle-ottawa Scale
OS: overall survival
PRISMA: the Preferred Reporting Items for Systematic Reviews and Meta-Analyses
PFS: progression-free survival
