# Supplementary material for: Prognostic significance of BRCA mutations in ovarian cancer: an updated systematic review with meta-analysis
Source: Oncotarget. 2016 Sep 28;8(1):285–302. doi: 10.18632/oncotarget.12306 (PMC5352118; doi:10.18632/oncotarget.12306)
Supplement: Supplementary file 1 [file oncotarget-08-285-s001.pdf]

Prognostic significance of *BRCA* mutations in ovarian cancer: an updated systematic review with meta-analysis

Supplementary Materials

Supplementary Table S1: Methodological quality of included studies based on the Newcastle–Ottawa Scale for included studies

Observational studies[a]

Study	Design	Selection	Comparability	Outcome/exposure	Overall quality (max 9)
Synowiec(2016)	Cohort	★★★	★★	★★★	8
Sabatier(2016)	Cohort	★★★	★★	★★★	8
Kotsopoulos(2016)	Cohort	★★★	★	★★★	7
Harter(2015)	Cohort	★★★	★★	★★★	8
Chen(2015)	Cohort	★★★	★	★★★	7
Candido-dos-Reis(2015)	Cohort	★★★	★★	★★★	8
Cunningham (2014)	Cohort	★★★	★★	★★★	8
Zhang(2014)	Cohort	★★★	★★	★★★	8
Rudaitis(2014)	Cohort	★★★	★★	★★★	8
Pennington(2014)	Cohort	★★★	★★	★★★	8
Safra(2013)	Cohort	★★★	★★	★★★	8
McLaughlin(2013)	Cohort	★★★	★★	★★★	8
Hyman(2012)	Cohort	★★★	★	★★★	7
Dann(2012)	Cohort	★★★	★	★★★	7
Chan(2012)	Cohort	★★★	★★	★★	7
Alsop(2012)	Cohort	★★★	★	★★★	7
Yang(2011)	Cohort	★★★	★★	★★★	8
Lacour(2011)	Cohort	★★★	★★	★★★	8
Gallagher(2011)	Cohort	★★★	★★	★★★	8
Hennessy(2010)	Cohort	★★★	★★	★★★	8
Tan(2008)	Case-control	★★★	★★	★★	7
Chetrit(2008)	Cohort	★★★	★★	★★★	8
Pal(2007)	Cohort	★★★	★★★	★★★	8
Majdak(2005)	Case-control	★★★	★★	★★	7
Cass(2003)	Cohort	★★★	★	★★★	7
David(2002)	Cohort	★★	★	★★★	6
Buller(2002)	Case-control	★★★	★★	★★	7
Zweemer(2001)	Case-control	★★★	★★	★★	7
Ramus(2001)	Cohort	★★★	★	★★★	7
Boyd(2000)	Cohort	★★★	★★	★★★	8
Pharoah(1999)	Case-control	★★★	★★	★★	7
Johannsson(1998)	Case-control	★★★	★★	★★	7
Aida(1998)	Case-control	★★★	★★	★★	7
Rubin(1996)	Case-control	★★★	★★	★★	7

[a]Study quality assessment of observational studies performed using the Newcastle–Ottawa scale (each asterisk represents if individual criterion within the subsection were fulfilled).

Supplementary Appendix S1: Search strategies

Search strategy for Pubmed (Publication date to 2016/02/09)

1. "Ovary"[Mesh]
2. "Neoplasms"[Mesh]
3. 1 AND 2
4. ovary[Title/Abstract] OR Ovarian[Title/Abstract] OR oophor*[Title/Abstract]
5. neoplas*[Title/Abstract] OR tumor*[Title/Abstract] OR tumour*[Title/Abstract]) OR cancer*[Title/Abstract] OR carcinoma*[Title/Abstract] OR malignan*[Title/Abstract] OR Neoplasms[Title/Abstract]
6. 4 AND 5
7. 3 OR 6
8. "Mutation"[Mesh]
9. mutation[Title/Abstract] OR mutation*[Title/Abstract] OR mutated[Title/Abstract]
10. 8 OR 9
11. "Genes, BRCA1"[Mesh]
12. "Genes, BRCA2"[Mesh]
13. brca1[Title/Abstract] OR brca2[Title/Abstract] OR brca-1[Title/Abstract] OR brca-2[Title/Abstract] OR brca[Title/Abstract] OR brca*[Title/Abstract])
14. 11 OR 12 OR 13
15. 14 AND 10
16. "Mortality"[Mesh]
17. "Survival"[Mesh]
18. "Prognosis"[Mesh]
19. prognos*[Title/Abstract] OR survival[Title/Abstract] OR recurren*[Title/Abstract] OR mortality[Title/Abstract] OR predict*[Title/Abstract] OR outcome*[Title/Abstract] OR death[Title/Abstract]
20. 16 OR 17 OR 18 OR 19
21. 7 AND 15 AND 20

Search strategy for Embase (Publication date to 2016/02/09)

1. 'ovary tumor'/exp
2. 'neoplasm'/exp
3. neoplas*:ab,ti OR tumor*:ab,ti OR tumour*:ab,ti OR cancer*:ab,ti OR carcinoma*:ab,ti OR malignan*:ab,ti OR neoplasms:ab,ti
4. 'ovary'/exp
5. ovarian:ab,ti OR ovary:ab,ti
6. #2 OR #3
7. #4 OR #5
8. #6 AND #7
9. #1 OR #8
10. 'brca1 protein'/exp
11. 'brca2 protein'/exp
12. brca1:ab,ti OR brca2:ab,ti OR 'brca 1':ab,ti OR 'brca 2':ab,ti OR brca:ab,ti OR brca*:ab,ti
13. 'mutation'/exp
14. mutation:ab,ti OR mutation*:ab,ti OR mutated:ab,ti
15. #10 OR #11 OR #12
16. #13 OR #14
17. #15 OR #16
18. 'mortality'/exp
19. 'death'/exp
20. 'survival'/exp
21. 'prediction'/exp
22. 'prognosis'/exp
23. mortality:ab,ti OR death:ab,ti OR survival:ab,ti OR predict*:ab,ti OR prognos*:ab,ti OR recurren*: ab,ti OR outcome*: ab,ti
24. #18 OR #19 OR #20 OR #21 OR #22 # 23
25. #9 AND #17 AND #25
